# Heterojunction of Silicon Nanowires and TiO_2_ via Bioinspired Polymer for Efficient Photocatalytic Hydrogen Evolution

**DOI:** 10.1021/acsami.5c03529

**Published:** 2025-05-29

**Authors:** Jakub Szewczyk, Stefanos Chaitoglou, Igor Iatsunskyi, Ghulam Farid, Mariusz Jancelewicz, Roger Amade-Rovira, Enric Bertran-Serra, Emerson Coy

**Affiliations:** † NanoBioMedical Centre, 529746 Adam Mickiewicz University, Wszechnicy Piastowskiej 3, 61-614 Poznan, Poland; ‡ Department of Applied Physics, 16724 University of Barcelona, C/Martí i Franquès, 1, 08028 Barcelona, Catalunya, Spain; § ENPHOCAMAT Group, Institute of Nanoscience and Nanotechnology (IN2UB), 16724 University of Barcelona, C/Martí i Franquès, 1, 08028 Barcelona, Catalunya, Spain

**Keywords:** bioadhesion, photoreduction, functionalization, band alignment, redox, heterointerface, nanocomposite

## Abstract

The use of photocatalytic hydrogen evolution depends on the availability of an efficient photocatalyst. This experiment aimed to create a type-II heterojunction between silicon nanowires and titanium dioxide bridged by a biomimetic polydopamine layer for efficient photocatalytic hydrogen production. This delivered adhesion, mechanical durability, outstanding wettability, and effective transfer of photogenerated charge carriers. Electron microscopy provided insight into the morphology and integration of the nanocoatings, while X-ray photoelectron spectroscopy confirmed the chemical structure of the nanocomposite before and after photocatalytic tests. These included voltammetric and impedance spectroscopy studies under UV–vis irradiation, which revealed a significant and stable photocurrent (up to 300 μA cm^–2^) and facilitated electron transfer through the hybrid photocatalyst. Finally, we composed a band diagram of the obtained remote heterojunction, which allowed us to understand its operation principles. We believe this approach provides a different perspective on type-II heterojunctions and allows for further development of the band alignment strategies.

## Graphical abstract

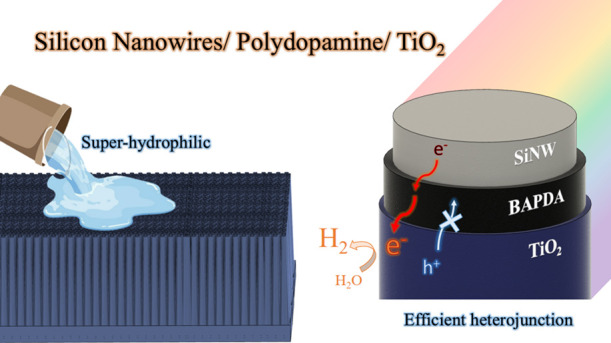

## Introduction

Silicon nanowires (SiNWs) produced by metal-assisted chemical etching (MACE) are attractive for industrial-scale heterogeneous photocatalysis due to their relatively low cost, high-yield synthesis techniques, and inherent high numbers of active sites.^1–3^ In the UV–vis-NIR region, the optical absorbance of SiNWs is one to 2 orders of magnitude higher than flat silicon.^4,5^ It was indeed shown that the SiNWs photoelectrode was more effective than flat Si for the photoelectrocatalytic hydrogen evolution reaction (HER). Specifically, the nanostructure of the nanowires provided a more effective surface area for the nucleation, growth, and quick release of hydrogen gas bubbles.^6^ However, using a silicon-based photoelectrode alone is insufficient for photocatalytic HER, due to nonoptimal band alignment, electron–hole recombination affinity, high overpotential required, poor chemical surface stability, and other drawbacks.^7–9^ For this reason, it needs to be combined with other cophotocatalysts, e.g., Ag^7^ and Pt^10^ nanoparticles, 2D materials—MoS_2_,^9–11^ rGO,^12^ and metal oxides.^13,14^ Practically, SiNW surfaces often show a hydrophobic character due to surface contamination and hydrogen termination upon chemical etching protocol or storage with exposure to the air.^15^ Therefore, oxygen plasma treatment or other surface treatment protocols are necessary to remove organic contaminants and increase the density of hydroxyl groups to restore hydrophilicity—an essential feature of the photocatalyst for photocatalytic water splitting.^16^

Here, we performed a SiNW-based photoelectrode modification with a hybrid coating of a polymer and titanium dioxide in this experiment. The biomimetic polymer we used was polydopamine, which has been extensively researched and described in the literature over the past few years.^17,18^ Polydopamine has unique electrochemical properties^19^ and a certain degree of supramolecular ordering,^20^ but most importantly, it is extensively used to improve various inorganic photocatalysts.^21^ Striving for a thin and conformal coating on the SiNW surface, we used the recently described modification of polydopamine with boric acid (BAPDA).^22,23^ It was shown that it can inhibit^24^ or entirely halt^25^ the formation of polydopamine. However, thoughtful BA-modified synthesis decelerates the dopamine oxidation process, facilitating the development of a thin, uniform layer with superior mechanical properties.^22^ Finally, due to its exceptionally robust adhesion and mechanical strength, it was able to provide an anchoring layer for a thin TiO_2_ film. This transition metal oxide has long been recognized as a prominent candidate for practical photocatalytic hydrogen production.^26^ Furthermore, studies have demonstrated that the deposition of an ultrathin layer (a few nm) onto silicon facilitates the formation of an efficient heterojunction.^27^ Atomic layer deposition (ALD) facilitates the controlled deposition of a TiO_2_ layer with a thickness of approximately 5 nm, which aligns with the characteristic size range of TiO_2_ nanoparticles exhibiting optimal photocatalytic activity.^28–30^ Therefore, we aimed to create a type-II heterojunction between SiNWs and titanium oxide bonded by a BAPDA layer to ensure adhesion and effective transfer of photogenerated charges.

## Results and Discussion

Transmission electron microscopy (TEM) characterization of the TiO_2_/BAPDA/SiNWs heterostructure provides insights into integrating nanometric coatings on the Si surface. An enlarged image of the SiNWs is shown in Figure 1a. The vertical alignment of the nanowires is lost during their transfer from the substrate to the Cu grip. Nevertheless, the approximate dimensions of the nanowires can be estimated. Two distinct sets of NWs are observed, the first featuring a diameter of ∼60 nm and the second featuring a diameter of ∼100 nm. The formation of NWs with varying diameters is an intrinsic feature of the preparation process. MACE is a top-down preparation approach, where Ag particles chemically etch Si in the presence of HF. Therefore, their size determines the etched area in silicon wafers and subsequently the diameter of the resulting Si NWs.^31^ Variations in the Ag particle diameter ultimately result in the formation of Si NWs with varying diameters. Bundling and aggregation of individual NWs may also be responsible for the wider nanostructures observed by TEM. The length of the nanowires is on the order of micrometers; nevertheless, precise values can not be estimated due to the damage of NWs rising from the transferring process. Magnified images provide information regarding the TiO_2_ and BAPDA coating deposition on the SiNWs and the resulting interface. The crystal planes of Si (111) are observed in the interface with the TiO_2_. The TiO_2_ thickness is ∼5 nm (Figure 1b). It is noted that the TiO_2_ layer is always present, while there are observed zones with (Figure 1c) and without (Figure 1b) the BAPDA film. This may arise from the distinct deposition techniques used—BAPDA was deposited onto SiNWs by dip-coating. Perhaps the reaction solution could not be evenly supplied over the entire surface of the SiNWs. However, the X-ray photoelectron spectroscopy (XPS) studies described later clearly confirmed BAPDA layers. When present, the BAPDA film thickness is ∼13 nm (Figure 1c).

**Figure 1:**
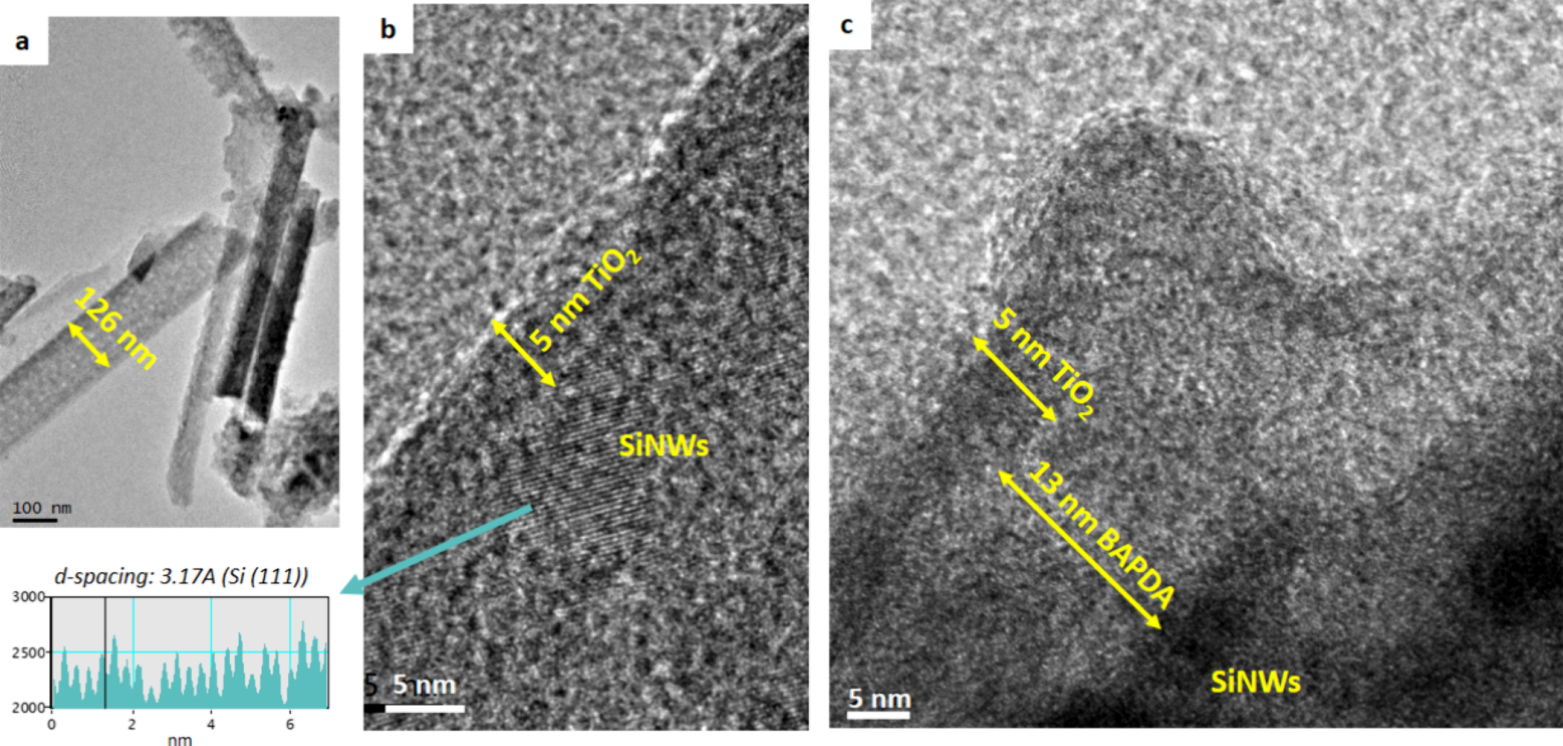
(a) TEM image of the SiNWs transferred on the Cu grid, (b) enlarged image of the TiO_2_/SiNWs interface in an area free of BAPDA, (c) enlarged image of the TiO_2_/BAPDA/SiNWs interface in an area with BAPDA.

SEM and EDS characterization of the as-prepared composite reveals its morphology, chemical composition, and distribution. The SiNWs are well aligned and perpendicularly oriented with respect to the Si substrate. Their length is ∼20 μm (Figure 2a). EDS elemental mapping shows the distribution of Si, Ti, and Ag. It is noted that Ti traces are observed across the whole length of the SiNWs; nevertheless, a denser distribution is observed on the top 2 μm. Interestingly, Ag trace is detected in the SiNWs/Si substrate interface, revealing its poor chemical etching (Figure 2b). An EDS spectrum with signal taken from the SiNWs area shows peaks related to Ti, Si, and O, excluding the presence of contaminants or residual species (Figure 2c).

**Figure 2:**
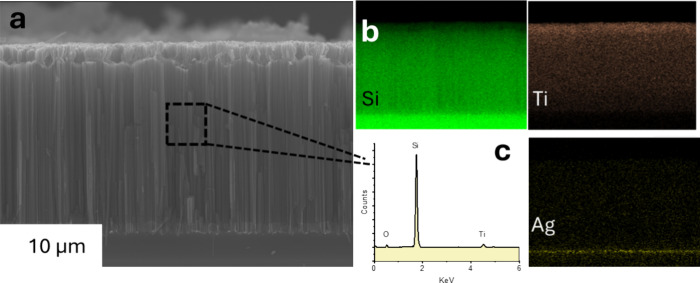
(a) SEM image of the as-prepared SiNWs on Si substrate, (b) elemental mapping of Si, Ti, and O, and (c) EDS spectrum of the as-prepared SiNWs.

We conducted XPS analysis to achieve two objectives: (1) to verify the presence of BAPDA and TiO_2_ thin layers chemically and (2) to examine their durability and stability following a series of photocatalytic tests. While these tests will be detailed later, it is crucial to consider that prolonged exposure to fluctuating potentials, intense radiation, and electrolyte interactions may degrade the BAPDA and TiO_2_ coatings. Therefore, we employ XPS to compare their composition before and after testing.

The C 1s spectrum (Figure 3a) indicates that carbon bonds typical of BAPDA are present, i.e., C=C, C–C, C–H (∼285.02 eV), C–O, C–N (∼286.80 eV), and C=O (∼289.31 eV), originating from various structural units,^22,23,25,32^ only very slight changes were noticed after photocatalytic tests. What is particularly important is that no significant loss was noted in the component containing C–H; this excludes the significant contribution of the C–H bond breaking in the photocatalytic evolution of free hydrogen.

**Figure 3:**
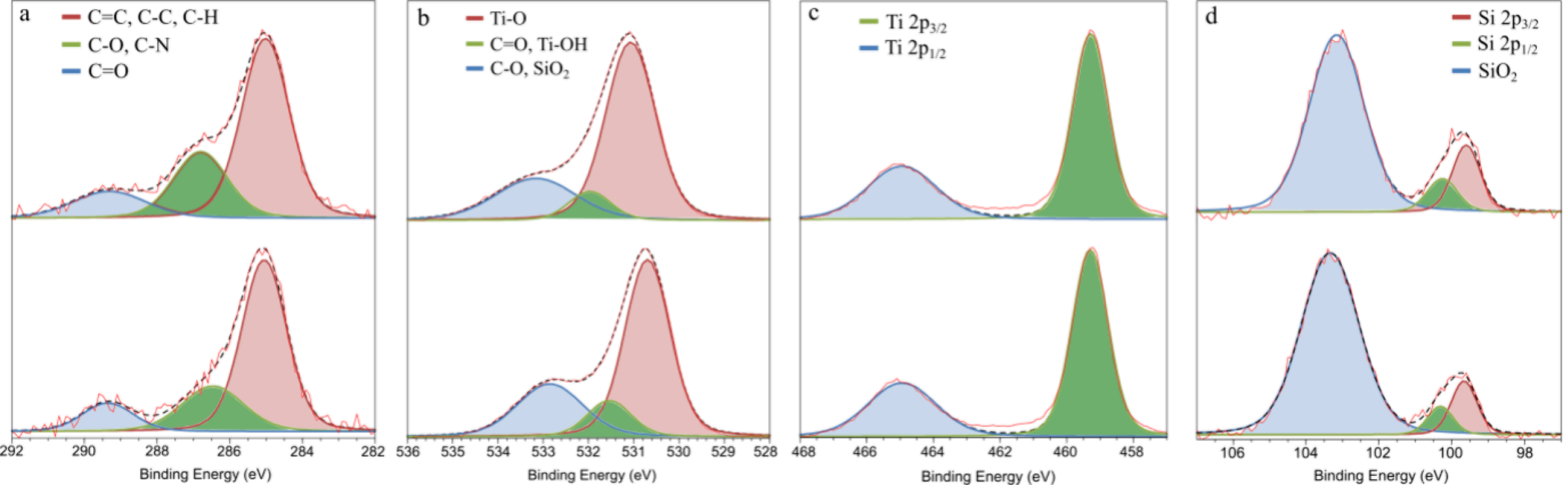
(a) XPS high-resolution spectra of the C 1s, (b) O 1s, (c) Ti 2p, and (d) Si 2p regions of the SiNWR/BAPDA/TiO_2_ sample before (up) and after (down) the photocatalytic tests.

Interestingly, the O 1s spectrum (Figure 3b) shows a slight oxidation of SiNWs by the increased SiO_2_ component (∼531.54 eV) due to long-term photocatalytic tests. This underlines the importance of the passivation protective layer. The titanium region on the XPS spectrum (Ti 2p) reveals Ti^4+^ energy levels exclusively (Figure 3c), which is characteristic of well-defined TiO_2_.^33,34^ Importantly, the lack of shift and reduction in the intensity indicates the robustness of the TiO_2_ for photocatalytic testing. Moreover, this likely indicates the absence of H^+^ ion intercalation, which can be attributed in part to the inherent stability of the TiO_2_ layer, as well as to the neutral pH conditions of the test.^35^

Finally, Si 2p (Figure 3d), which shows mainly SiO_2_ components, has a slightly less intense signal originating from Si itself. The SiNWs are covered with TiO_2_ and BAPDA, while silicon oxidation occurs on the surface. Nevertheless, the SiNW/BAPDA/TiO_2_ structure is hereby confirmed. The information about the peaks and their assignments is summarized in Table 1 below. We also supplement the XPS full spectra of the SiNW/BAPDA/TiO_2_ sample in Figure S1.

**Table 1:** XPS High-Resolution Spectra before and after Photocatalytic Tests—Binding Energies, FWHM Values, and Area Percentage

spectrum	peak	binding energy (eV)	FWHM	area %
C 1s before test	C=C, C–C, C–H	285.02	1.55	60.6
	C–O, C–N	286.80	1.76	25.5
	C=O	289.31	1.46	13.9
C 1s after test	C=C, C–C, C–H	285.05	1.45	64.3
	C–O, C–N	286.47	2.01	23.5
	C=O	289.36	1.67	12.2
O 1s before test	Ti–O	530.69	1.13	65.9
	C=O, Ti–OH	532.52	1.83	25.09
	C–O, SiO_2_	531.47	0.95	8.98
O 1s after test	Ti–O	530.70	1.17	61.4
	C=O, Ti–OH	532.85	1.71	26.5
	C–O, SiO_2_	531.54	1.14	12.1
Ti 2p before test	Ti 2p_3/2_	459.29	1.29	64.9
	Ti 2p_1/2_	464.94	2.43	35.1
Ti 2p after test	Ti 2p_3/2_	459.30	1.28	65.0
	Ti 2p_1/2_	464.94	2.41	35.0
Si 2p before test	SiO_2_	103.16	1.78	77.1
	Si 2p_3/2_	100.26	0.99	8.1
	Si 2p_1/2_	99.60	0.90	14.8
Si 2p after test	SiO_2_	103.33	1.88	83.4
	Si 2p_3/2_	100.31	0.82	5.7
	Si 2p_1/2_	99.66	0.83	10.9

After confirming the successful deposition of the BAPDA and TiO_2_ thin films, we performed the water contact angle test (Figure S2). As expected, the sample surface changed from hydrophobic in the case of SiNW (132 ± 7°) to superhydrophilic in the case of SiNW/BAPDA/TiO_2_ (5.7 ± 0.5°). This had a great impact on the photocatalytic efficiency, as described below.

We started photocatalytic tests with electrochemical impedance spectroscopy (EIS) in the dark and under UV–vis irradiation (Figure 4a). The following EEC (Table S1) represented our system well: 4 typical R/CPE elements connected in series: three R/CPE in the high-frequency region and one R/CPE in the low-frequency region. The n factor in each CPE was close enough to good capacitance representation—the use of CPE was necessary only for good fitting, not to represent some unusual processes or phenomena.^36^ The first three R/CPEs likely correspond to (1) resistance and capacitance of the semiconductor/electrolyte interface, (2) surface states or defect states in the semiconductor, and (3) charge carrier transport resistance within the semiconductor bulk.^36,37^ It should be noted that in the high frequency range, the Q_1_ and Q_3_ (CPE) values for SiNW are significantly lower than for SiNW/BAPDA/TiO_2_, which indicates more rapid charge carrier dynamics and more negligible capacitive and faradaic processes.^36^ The charge transfer mechanism in SiNW/BAPDA/TiO_2_ may be more complex, as will be discussed later. The resistance of all these components undergoes a reduction upon UV–vis light, both for SiNW and for SiNW/BAPDA/TiO_2_. This is particularly visible for R_3_ in the SiNW/BAPDA/TiO_2_ sample: the resistance is reduced by over 7 times. The deposition of BAPDA and TiO_2_ coatings probably creates a favorable band alignment that accelerates the transport of charge carriers in bulk nanowires through the resulting internal electric field. This will be explained in more detail later. The fourth R/CPE should be typically related to mass transport limitations (e.g., diffusion of reactants in the electrolyte).^36,37^ The resistance of this component is also significantly lower for SiNW/BAPDA/TiO_2_ than for pristine SiNW. The reason for this is probably the previously mentioned super hydrophilicity, which facilitates the transport of molecules to/from the photocatalyst surface in an aqueous environment. Cyclic voltammetry (CV) proved that, unlike SiNW itself, the SiNW/BAPDA/TiO_2_ nanocomposite exhibits a significant photocurrent response to UV–vis light (Figure 4b). Within the oxidation scan, the response is not clear—the electrode tends to polarize negatively, which reduces the generated positive current. However, in the reduction scan, the negative polarization additionally enhances the already very significant negative peak, which makes the photocurrent response impressive and proposes that SiNW/BAPDA/TiO_2_ is well suited for the photocatalytic HER. This is confirmed with linear sweep voltammetry (LSV) under chopped UV–vis illumination (Figure 4c). Note that the photocurrent generated by SiNW/BAPDA/TiO_2_ over the entire investigated range is significant. It reaches up to ∼300 μA cm^–2^ at the potential equal −0.6 V vs NHE. However, even with a relatively low potential vs hydrogen production (inset image), it reaches ∼100 μA cm^–2^. The question arises whether such a good result would be stable over time. Therefore, we performed chronoamperometry (CA) under chopped UV–vis (Figure 4d) for 1 h. The potential versus NHE at which we performed this examination was −0.21 V, in order to be close to real HER conditions. For the first ∼10 min, the photogenerated current slowly decreases, but then it stabilizes. As a result, after 1 h the photocurrent is approximately 70% of what it was after 10 min (Figure 4e,f). It is worth emphasizing that each light on/off cycle lasts only 1 s; therefore, 1 h of the experiment corresponds to 1800 light on/off cycles.

**Figure 4:**
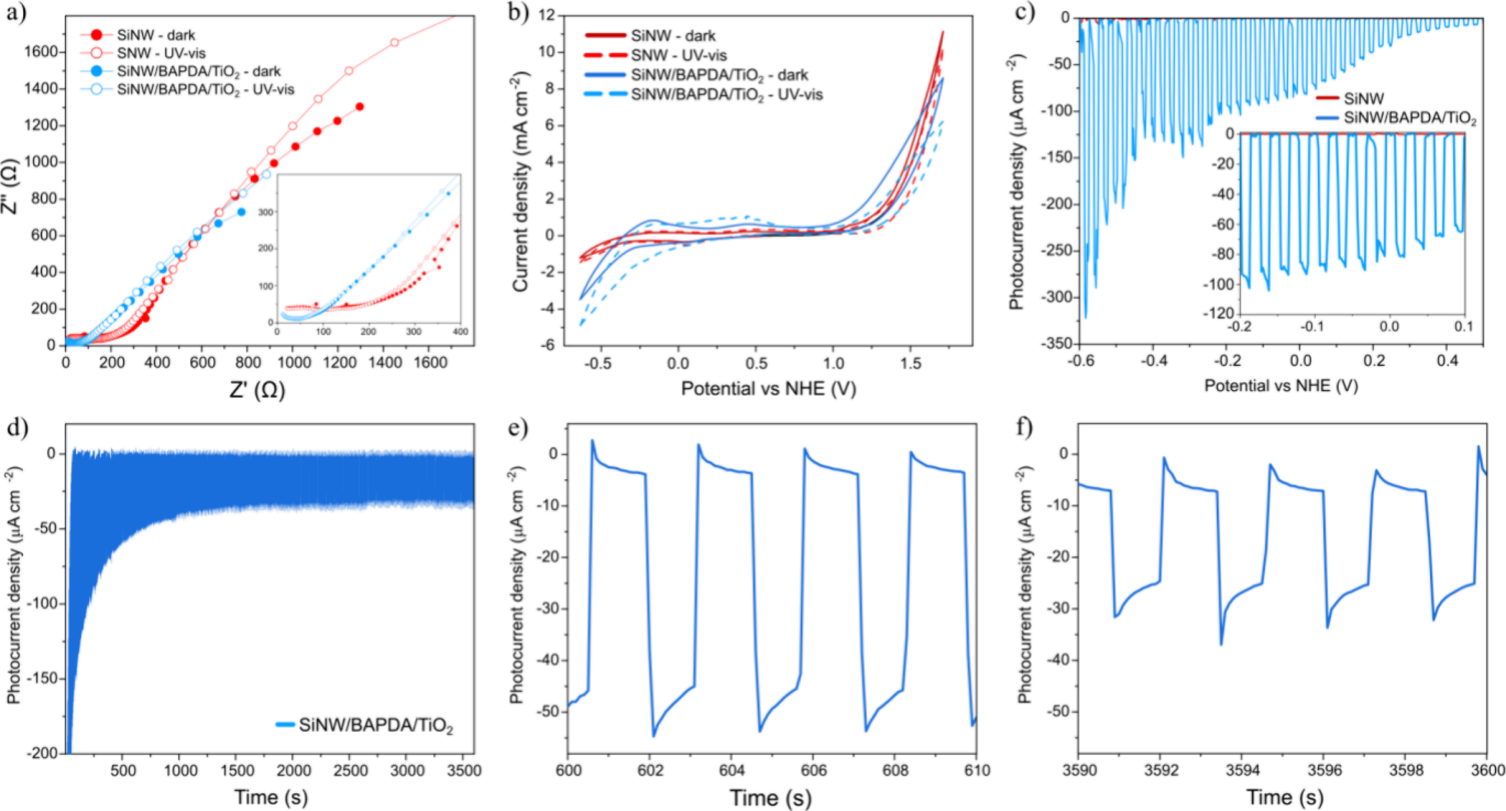
Photocatalytic characterization of the SiNW and SiNW/BAPDA/TiO_2_ in the dark and UV–vis: (a) electrochemical impedance spectroscopy (inset: zoom to the high-frequency region), (b) cyclic voltammetry, (c) linear sweep voltammetry under chopped irradiation (current in the dark was subtracted), (d) chronoamperometry under chopped irradiation (current in the dark was subtracted), (e) zoom to 10 min, (f) zoom to 1 h.

It is recognized that SiNWs have exceptionally high absorbance in the broad UV–vis range,^38^ which is desirable from the point of view of photocatalytic applications. According to the literature, the bandgap of SiNWs with diameters of a few nanometers can increase to as much as 3.5 eV, as quantum confinement effects become significant.^39,40^ However, for diameters on the order of several tens of nanometers, the bandgap value remains characteristic of bulk silicon, approximately 1.1–1.2 eV.^41^ The question arises whether BAPDA and TiO_2_ coatings did not significantly reduce the UV–vis absorbance, which would be unfavorable. The TiO_2_ bandgap is about 3.1–3.2 eV^20,42^ and could contribute to the reflection of a significant amount of visible light incident on the sample. Therefore, we measured the UV–vis diffuse reflectance and calculated the absorbance as 1-R, as shown in Figure 5a. Indeed, in the tested range (300–800 nm), SiNW shows outstanding ∼100% absorbance. Importantly, the TiO_2_ coating did not significantly reduce absorbance, even in the visible range, it does not fall short 80%. The UV–vis diffuse reflectance spectroscopy data facilitated the plotting of a Tauc plot for SiNW/BAPDA/TiO_2_ (Figure 5b). The measured band gap value was ∼2.4 eV, a significant reduction compared to the reference data of a pure TiO_2_.^20,42^ First, it was already shown that the polydopamine/TiO_2_ interface significantly influences the optical properties of the latter.^21,23,37,43,44^ Second, we postulate that we successfully created a heterojunction between silicon and titanium dioxide, even though they were separated by more than 10 nm by the BAPDA intermediate layer. The formation of a type II heterojunction between these semiconductors should lead to the shift in the flat band potential, which would partially explain the previously described EIS results. Such information can be obtained by comparing the Mott–Schottky EIS results for SiNW (Figure S3) and for SiNW/BAPDA/TiO_2_ (Figure 5c). The flat band potential versus NHE was shifted from 0.3 V for SiNW to −0.4 V for SiNW/BAPDA/TiO_2_.

**Figure 5:**
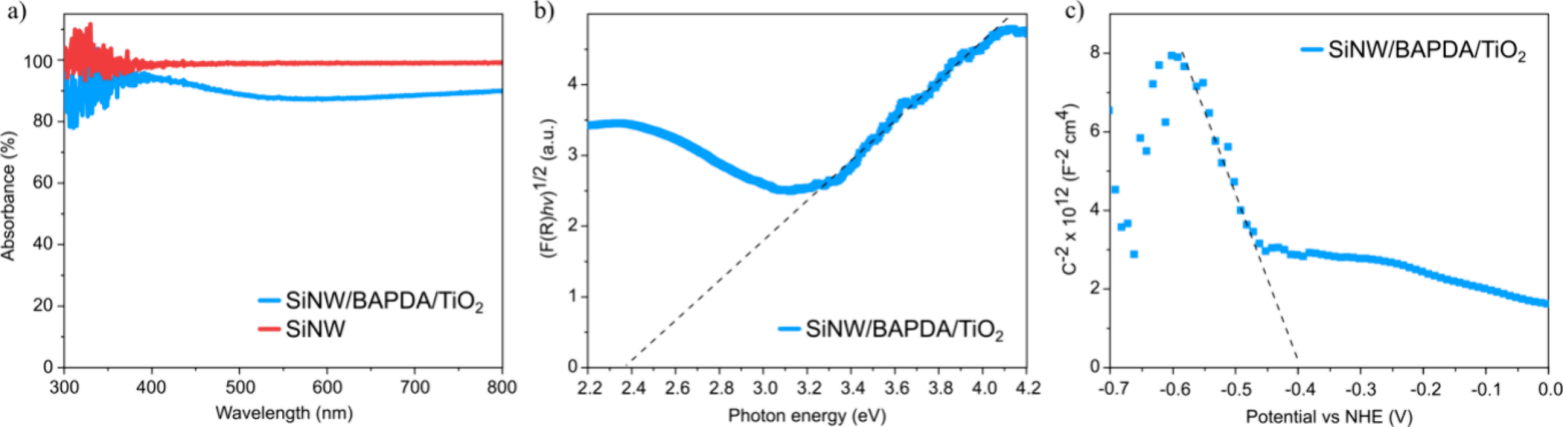
UV–vis diffuse reflectance spectroscopy of the SiNW and SiNW/BAPDA/TiO_2_: (a) absorbance spectra, (b) Tauc plot for the SiNW/BAPDA/TiO_2_, and (c) EIS Mott–Schottky analysis for the SiNW/BAPDA/TiO_2_.

The band diagram is crucial for explaining the phenomena described above (Figure 6a). BAPDA is not only a great interlayer—providing adhesion and chemical resistance—but it can also transport photogenerated charge carriers^37^ and extend their lifetimes.^23^ Its complex chemical structure gives rise to a multitude of energy levels, which originate from the molecular orbitals associated with the various chemical bonds.^45^ Of course, the actual role of BAPDA is more complex to elucidate since it is considered an amorphous semiconductor with a range of energy levels.^45,46^ Despite this, studies clearly show that it does not isolate the SiNW and TiO_2_ layers, which are not in direct physical contact, but rather facilitates the transport of their photogenerated charge carriers. As we know from calculations,^47,48^ the difference in the potentials of the conduction bands is significantly smaller than the difference in the potentials of the valence bands for silicon and titanium dioxide. Thus, electrons migrate easily across the type-II heterojunction, contributing to the negative polarization of the electrode and generating a strong photocurrent, facilitating a reduction reaction. Notably, electron–hole recombination across an S-scheme heterojunction would be expected to suppress the HER activity of the TiO_2_ surface while potentially enhancing oxygen evolution reaction activity by retaining holes with significant redox potential at the surface. However, as previously discussed, our observations indicate the opposite behavior. This does not imply that positive charges are incapable of migrating; however, their movement would predominantly be directed from TiO_2_ toward SiNW. In Figure 6b, we show schematically how the excited electron migrates from the bulk of the nanowire toward the TiO_2_ reactive surface.

**Figure 6:**
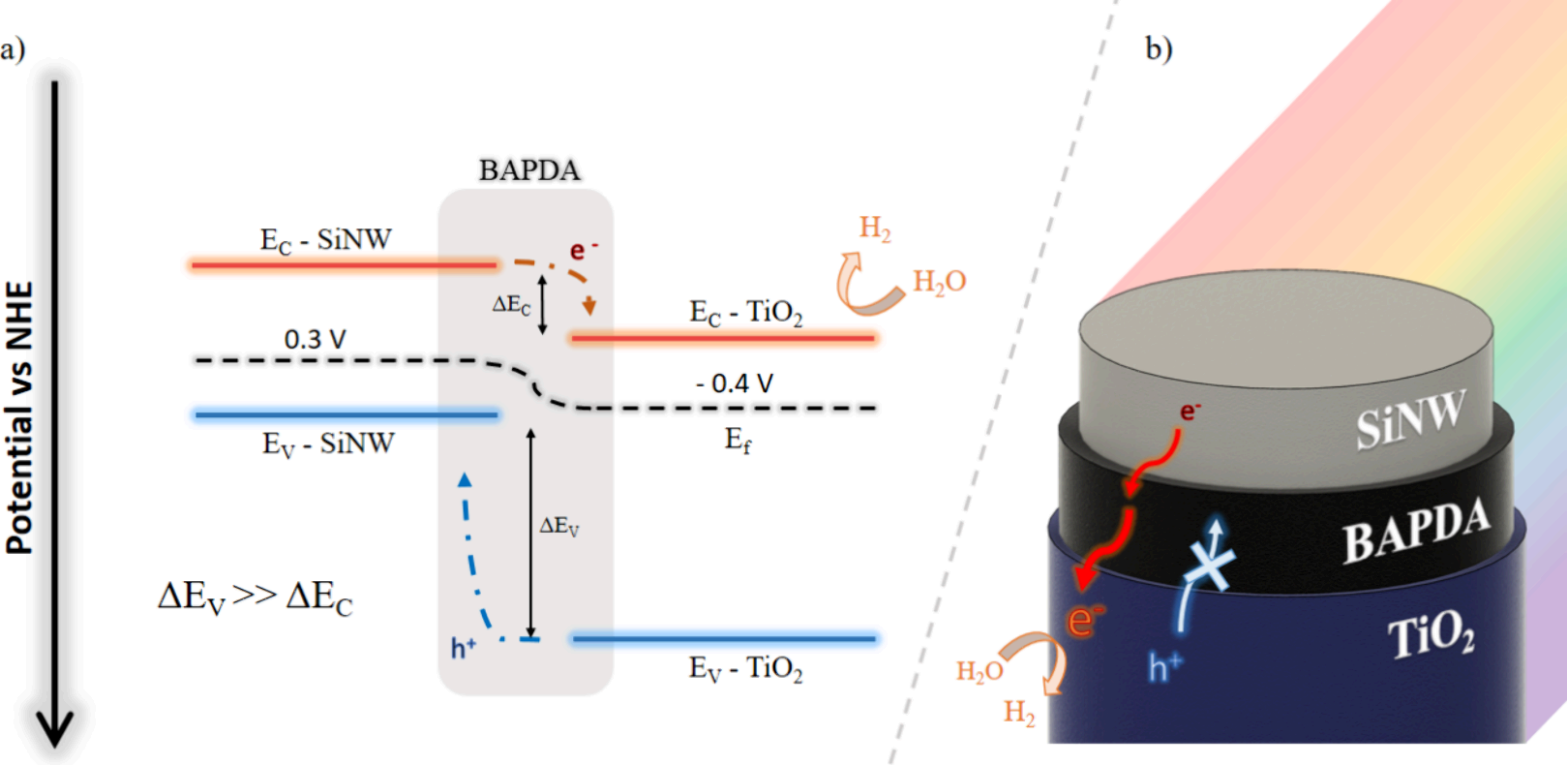
(a) Scheme of the band alignment in the heterojunction and (b) scheme of the photogenerated charge carrier migration paths in the nanocomposite.

## Conclusions

Using surface engineering, we were able to obtain SiNWs on a p-doped silicon wafer coated with a thin layer (∼13 nm) of biomimetic polydopamine (modified with boric acid) and titanium dioxide (∼5 nm). The resulting SiNW/BAPDA/TiO_2_ nanocomposites exhibited outstanding superhydrophilicity (5.7 ± 0.5°), without any surface activation or additional treatment. They provided very efficient photocatalytic reduction due to the BAPDA-mediated type-II heterojunction between SiNW and TiO_2_. Using 100 mW cm^–2^ UV–vis light, they produced a photocurrent up to 100 μA cm^–2^ at the HER potential or even ∼300 μA cm^–2^ at a more negative potential (−0.6 V vs NHE). Moreover, they generated very stable photocurrent—during 1800 light on/off cycles, its value dropped by only ∼30%. Our work provides a different perspective on type-II heterojunctions, allows for the further development of the band alignment strategies, and shows that the potential of polydopamine for photocatalysis and surface modification is enormous, promising a breakthrough in the field of photocatalytic hydrogen production beyond the laboratory scale.

## Methods

### SiNWs Preparation

SiNW arrays were prepared using MACE, following a procedure described elsewhere.^2^ The starting material for this process was commercially available p-type boron-doped silicon wafers (resistivity: 0.01–0.02 Ω cm from Si-Mat Germany). The wafers were thoroughly cleaned through a series of steps, beginning with ultrasonic cleaning in acetone and ethanol, followed by immersion in a hot solution of sulfuric acid (H_2_SO_4_) and hydrogen peroxide (H_2_O_2_), to ensure the removal of impurities. The fabrication process is divided into two main phases. The first phase involves the deposition of silver nanoparticles (AgNPs) on the silicon wafer surface. This was achieved by mixing a solution containing 0.02 M silver nitrate (AgNO_3_) and 5 M hydrofluoric acid (HF) at a 1:10 volume ratio. The AgNPs serve as catalysts for the formation of SiNW arrays. In the second phase, the silicon wafer coated with AgNPs underwent etching in a solution composed of 5 M HF and 30% H_2_O_2_, at a 10:1 volume ratio. This etching process is anisotropic, meaning it selectively etches the silicon surface in a way that results in vertically aligned SiNWs. Afterward, the samples were thoroughly rinsed and dried to remove any remaining chemicals. Additional treatments with 65% nitric acid (HNO_3_) and 2% hydrofluoric acid (HF) were performed to remove any residual silver nanoparticles and oxide layers, further enhancing the purity and quality of the SiNW arrays.

### Boric Acid Modified Polydopamine (BAPDA) Layer Preparation

Dopamine hydrochloride (DA) in concentration 0.5 mg mL^–1^ was added to a beaker containing tris buffer (10 mM, 100 mL, pH = 8.0) and an (anti)­oxidation agent, boric acid (BA). Due to our previous experience, the molar ratio of DA:BA was equal 1:3. Our objective was not to entirely inhibit the formation of polydopamine^25^ but rather to decelerate this process, thereby enhancing the quality of the deposited coating while minimizing contamination from dopamine oxidation byproducts.^22,23^ Previously, plasma-activated SiNWs samples were placed vertically in the solution by using hooks. The deposition took 4 h, which, according to our preliminary tests, should give a BAPDA thickness of approximately 10–15 nm. This was later confirmed by TEM and XPS. After 4 h, the samples were removed from the solution and hung on hooks to air-dry without additional treatment.

### Titanium Dioxide (TiO_2_) Coating Deposition

ALD was performed with the Picosun reactor, using TiCl_4_ and water as precursors, while nitrogen (N_2_) served both as a carrier and as a purging gas. The TiCl_4_ and water precursor containers were not heated, and evaporation occurred at room temperature. The TiO_2_ thin films were grown at a temperature of 200 °C in order to obtain an amorphous conformal coating. The growth rate was typically between 0.5 Å/cycle, and the number of cycles was 100, corresponding to approximately 5 nm final layer thickness. The growth was controlled by measuring the thickness of the film on Si-wafer reference substrates placed in the same reactor during the deposition process. Finally, the coating thickness and its amorphous nature were also assessed by using TEM (as described later).

### Chemical and Morphological Characterization

XPS analysis was performed with the PHI 5500 Multi-Technique System (from Physical Electronics, Chanhassen, MN, USA) using a monochromatic X-ray source (Al Kα line of 1486.6 eV and 350W. The area analyzed had a diameter of 0.8 mm, with Survey XPS spectra having a pass energy of 187.5 and 0.8 eV/step and elemental spectra having a pass energy of 11.75 and 0.1 eV/step. The software CasaXPS (version 2.3.25) was used for detailed high-resolution peak analysis involving fitting with Gaussian–Lorentzian functions. The charge correction of the spectra was done by setting the peak position of the C 1s main fitting component associated with the C–C bond to 285.00 eV and shifting all spectra accordingly.^49,50^

Morphological assessments were carried out using field emission scanning electron microscopy (FE-SEM) employing a JEOLJ-7100FE instrument and TEM using a JEOL 2100 instrument. The nanostructures were transferred onto a Cu grid by gently applying pressure with a cutter to detach them from the growth substrate.

The hydrophobic/hydrophilic nature of the surfaces was examined with an Ossila Water Contact Angle Goniometer. For each sample, at least 10 measurements were performed to enable a statistical analysis of the results. An ultrapure MiliQ water droplet (5 μL) was transferred onto the surface with pipet.

### Optical and Photocatalytic Characterization

UV–vis diffuse reflectance spectroscopy was performed with the Integrating Sphere (Ocean Optics Inc.) with a built-in UV–vis light source with an effective operational limit at approximately 350 nm. For data acquisition, an AvaSpec-Mini2048CL spectrometer (Avantes) was used. Acquisition time was 10 ms, and the number of averages was 50. Obtained spectra were transformed according to the Kubelka–Munk theory^51^ and plotted against the photon energy *hv.* ^52^ Absorbance was calculated as 100% – Reflectance, as the light Transmittance for our samples in the UV–vis range was close to 0%, and the scattering was eliminated by employing the integrating sphere. During all photocatalytic experiments, the light irradiation was sourced from a 300 W Xe arc lamp of a tunable light source (ScienceTech Inc.) with a spectral range of 300–1800 nm. It was calibrated at a 100 mW cm^–2^ power density. For EIS, CV, LSV, and CA data acquisition, a Gamry Reference 620 potentiostat (Gamry Instruments Inc.) in a three-electrode system was used. The investigated samples were a working electrode, Ag/AgCl/3 M KCl was a reference electrode, and a graphite rod was a counter electrode. The electrolyte used was 0.5 M Na_2_SO_4_ solution (pH = 6.5), saturated with gaseous nitrogen overnight and with argon for 1h before the experiments to exclude the influence of the dissolved oxygen. For each measurement, system conditioning was applied for at least 100 s. In the chopped light illumination experiments (LSV, CA), the light/dark cycle length was approximately 1 s, and the current of the system in the dark was subtracted from the graph by subtracting the baseline from the dark current value. The potential was converted from Ag/AgCl/3 M KCl to NHE using formula [Disp-formula eq1]:

$$E_{\mathrm{NHE}}\;(V)=E_{\mathrm{Ag}/\mathrm{AgCl}/3M\mathrm{KCl}}\;(V)+0.205\;(V)+0.059\times \mathrm{pH}\;(V)$$ 1

For the EIS EEC analysis, Z-View software was used. The chosen type of fitting was Complex, and the type of weighting was Calc-Modulus. For Mott–Schottky EIS analysis, measurements were performed with a 400 Hz frequency.

## Supplementary Material



## Data Availability

The data that support the findings of this study are available at: https://doi.org/10.5281/zenodo.14875733. Data that has not been made available in open access might be shared upon reasonable request to the corresponding author.

## Notes

X-ray photoelectron spectroscopy full spectra of the SiNW/BAPDA/TiO_2_ sample, water contact angle test results of the SiNW and SiNW/BAPDA/TiO_2_, electrochemical impedance spectroscopy equivalent electrochemical circuits—fitting model and parameters, and electrochemical impedance spectroscopy Mott–Schottky analysis for the SiNW (PDF)

## References

[1] Ray U., Sarkar S., Banerjee D. (2023). Silicon Nanowires as an Efficient
Material for Hydrogen Evolution through Catalysis: A Review. Catal. Today.

[2] Farid G., Amade-Rovira R., Ma Y., Ospina R., Serafin J., Chaitoglou S., Majumdar S., Poveda A., Bertran-Serra E. (2024). Improving
Lithium-Ion Battery Performance through Patterned Growth of Carbon
Nanotubes over Vertically Aligned Silicon Nanowires. J. Energy Storage.

[3] Ming T., Hu X., Wang Z., Wu X., Zuo X. (2024). The Nature of Photocatalytic
Hydrogen Generation on Silicon Nanowires Prepared by MAWC. Int. J. Hydrogen Energy.

[4] Tsakalakos L., Balch J., Fronheiser J., Korevaar B. A., Sulima O., Rand J. (2007). Silicon Nanowire Solar
Cells. Appl. Phys. Lett..

[5] Jung J.-Y., Guo Z., Jee S.-W., Um H.-D., Park K.-T., Lee J.-H. (2010). A Strong
Antireflective Solar Cell Prepared by Tapering Silicon Nanowires. Opt. Express.

[6] Feng R., Liu Y., Li S., Chen H., Song C., Tao P., Wu J., Zhang P., Deng T., Shang W. (2018). Hydrogen Evolution
from Silicon Nanowire Surfaces. RSC Adv..

[7] Ming T., Dietzek-Ivanšić B., Lu X., Zuo X., Sivakov V. (2022). Silicon Nanowires Decorated with
Silver Nanoparticles
for Photoassisted Hydrogen Generation. ACS Appl.
Energy Mater..

[8] Shen X., Sun B., Yan F., Zhao J., Zhang F., Wang S., Zhu X., Lee S. (2010). High-Performance Photoelectrochemical Cells from Ionic
Liquid Electrolyte in Methyl-Terminated Silicon Nanowire Arrays. ACS Nano.

[9] Younan S. M., Li Z., Fairchild M. P., Williams N. B., Huang Y., Gu J. (2022). Improving
the Stability of Silicon Nanowires During Photoelectrochemical Hydrogen
Generation with Zinc 1T-Phase Molybdenum Disulfide. Adv. Mater. Interfaces.

[10] Hsieh S. H., Ho S. T., Chen W. J. (2016). Silicon Nanowires with MoS and Pt
as Electrocatalysts for Hydrogen Evolution Reaction. J. Nanomater..

[11] Qiao L., Liao M., Fang K., He X., Zhang Y. (2019). Enhancement
of Photoelectrochemical Hydrogen Evolution of P-Type Silicon Nanowires
Array by Loading MoS_2_. Silicon.

[12] Sim Y., John J., Moon J., Sim U. (2018). Photo-Assisted Hydrogen
Evolution with Reduced Graphene Oxide Catalyst on Silicon Nanowire
Photocathode. Applied Sciences..

[13] Pavlenko M., Siuzdak K., Coy E., Załęski K., Jancelewicz M., Iatsunskyi I. (2020). Enhanced Solar-Driven
Water Splitting
of 1D Core-Shell Si/TiO_2_/ZnO Nanopillars. Int. J. Hydrogen Energy.

[14] Graniel O., Fedorenko V., Viter R., Iatsunskyi I., Nowaczyk G., Weber M., Załęski K., Jurga S., Smyntyna V., Miele P., Ramanavicius A., Balme S., Bechelany M. (2018). Optical Properties
of ZnO Deposited
by Atomic Layer Deposition (ALD) on Si Nanowires. Mater. Sci. Eng., B.

[15] Yilbas B. S., Salhi B., Yousaf M. R., Al-Sulaiman F., Ali H., Al-Aqeeli N. (2016). Surface Characteristics of Silicon Nanowires/Nanowalls
Subjected to Octadecyltrichlorosilane Deposition and n-Octadecane
Coating. Sci. Rep..

[16] Li A., Zhang P., Kan E., Gong J. (2024). Wettability Adjustment
to Enhance Mass Transfer for Heterogeneous Electrocatalysis and Photocatalysis. eScience.

[17] Lee H., Dellatore S. M., Miller W. M., Messersmith P. B. (2007). Mussel-Inspired
Surface Chemistry for Multifunctional Coatings. Science..

[18] Ball V. (2024). Polydopamine
Films: Versatile but Interface-Dependent Coatings. Nanotechnol. Rev..

[19] Szewczyk J., Aguilar-Ferrer D., Coy E. (2022). Polydopamine Films: Electrochemical
Growth and Sensing Applications. Eur. Polym.
J..

[20] Szewczyk J., Radhakrishnan D., Łukasiewicz Z., Coy E. (2024). Review on Polydopamine
Supramolecular Ordering–Mechanism Elucidation and Application
in 2D Nanocomposites Fabrication. Eur. Polym.
J..

[21] Aguilar-Ferrer D., Szewczyk J., Coy E. (2022). Recent Developments
in Polydopamine-Based
Photocatalytic Nanocomposites for Energy Production: Physico-Chemical
Properties and Perspectives. Catal. Today.

[22] Szewczyk J., Babacic V., Krysztofik A., Ivashchenko O., Pochylski M., Pietrzak R., Gapiński J., Graczykowski B., Bechelany M., Coy E. (2023). Control of Intermolecular
Interactions toward the Production of Free-Standing Interfacial Polydopamine
Films. ACS Appl. Mater. Interfaces.

[23] Szewczyk J., Tjardts T., Symalla F., Iatsunskyi I., Faupel F., Aktas C., Coy E., Veziroglu S. (2025). Boric Acid
Modified Polydopamine and Nanocolumnar Hydrogenated TiO_2_ Nanocomposite with Improved Photocatalytic Performance. Appl. Surf. Sci..

[24] Huang C., Wang X., Yang P., Shi S., Duan G., Liu X., Li Y. (2023). Size Regulation of
Polydopamine Nanoparticles by Boronic
Acid and Lewis Base. Macromol. Rapid Commun..

[25] Schneider A., Hemmerlé J., Allais M., Didierjean J., Michel M., D’Ischia M., Ball V. (2018). Boric Acid as an Efficient
Agent for the Control of Polydopamine Self-Assembly and Surface Properties. ACS Appl. Mater. Interfaces.

[26] Rafique M., Hajra S., Irshad M., Usman M., Imran M., Assiri M. A., Ashraf W. M. (2023). Hydrogen
Production Using TiO2-Based
Photocatalysts: A Comprehensive Review. ACS
Omega.

[27] Yao X., Chen L., Liu M., Feng D., Wang C., Lu F., Wang W., Wang X., Cheng Y., Liu H., Chen H., Wang W. (2018). Rational Design of Si/TiO2 Heterojunction
Photocatalysts: Transfer Matrix Method. Appl.
Catal. B Environ..

[28] Egli̅tis R., Joost U., Zukuls A., Rubenis K., Ignata̅ns R., Avotiṇa L., Baumane L., Šmits K., Hirsimäki M., Käämbre T., Šutka A. (2020). Strong, Rapid,
and Reversible Photochromic Response of Nb Doped TiO2 Nanocrystal
Colloids in Hole Scavenging Media. ACS Appl.
Mater. Interfaces.

[29] Iesalnieks M., Egli̅tis R., Juhna T., Šmits K., Šutka A. (2022). Photocatalytic Activity of TiO2 Coatings Obtained at
Room Temperature on a Polymethyl Methacrylate Substrate. Int. J. Mol. Sci..

[30] Joost U., Šutka A., Oja M., Smits K., Döbelin N., Loot A., Järvekülg M., Hirsimäki M., Valden M., Nõmmiste E. (2018). Reversible Photodoping of TiO2 Nanoparticles
for Photochromic Applications. Chem. Mater..

[31] Farid G., Amade-Rovira R., Ma Y., Chaitoglou S., Ospina R., Bertran-Serra E. (2024). Revolutionizing Energy Storage: Silicon
Nanowires (SiNWs) Crafted through Metal-Assisted Chemical Etching. Arab. J. Chem..

[32] Hemmatpour H., De Luca O., Crestani D., Stuart M. C. A., Lasorsa A., van der Wel P. C. A., Loos K., Giousis T., Haddadi-Asl V., Rudolf P. (2023). New Insights in Polydopamine Formation via Surface
Adsorption. Nat. Commun..

[33] Biesinger M. C., Lau L. W. M., Gerson A. R., Smart R. S. C. (2010). Resolving
Surface
Chemical States in XPS Analysis of First Row Transition Metals, Oxides
and Hydroxides: Sc, Ti, V. Cu and Zn. Appl.
Surf. Sci..

[34] Szewczyk J., Iatsunskyi I., Michałowski P.
P., Załęski K., Lamboux C., Sayegh S., Makhoul E., Cabot A., Chang X., Bechelany M., Coy E. (2024). TiO_2_/PDA
Multilayer Nanocomposites with Exceptionally Sharp Large-Scale Interfaces
and Nitrogen Doping Gradient. ACS Appl. Mater.
Interfaces.

[35] Iesalnieks M., Vanags M., Alsiṇa L. L., Egli̅tis R., Gri̅nberga L., Sherrell P. C., Šutka A. (2024). Efficient
Decoupled Electrolytic Water Splitting in Acid through Pseudocapacitive
TiO_2_. Adv. Sci..

[36] Lazanas A. C., Prodromidis M. I. (2023). Electrochemical Impedance Spectroscopy—A
Tutorial. ACS Meas. Sci. Au.

[37] Szewczyk J., Ziółek M., Siuzdak K., Iatsunskyi I., Pochylski M., Aguilar-Ferrer D., Kempiński M., Tanos F., Gapiński J., Bechelany M., Coy E. (2024). Ex-Situ Transferring of Polydopamine
Films on Semiconductor Interface:
Evidence of Functional Hybrid Heterojunction. Eur. Polym. J..

[38] Hutagalung S. D., Fadhali M. M., Areshi R. A., Tan F. D. (2017). Optical and Electrical
Characteristics of Silicon Nanowires Prepared by Electroless Etching. Nanoscale Res. Lett..

[39] Holmes J. D., Johnston K. P., Doty R. C., Korgel B. A. (2000). Control of Thickness
and Orientation of Solution-Grown Silicon Nanowires. Science..

[40] Ma D. D. D., Lee C. S., Au F. C. K., Tong S. Y., Lee S. T. (2003). Small-Diameter
Silicon Nanowire Surfaces. Science..

[41] Kurokawa Y., Yano M., Miyajima S., Yamada A. (2017). Bandgap Tuning of Silicon
Nanowire Arrays for Application to All-Silicon Tandem Solar Cells. Jpn. J. Appl. Phys..

[42] Nakata K., Fujishima A. (2012). TiO2 Photocatalysis:
Design and Applications. J. Photochem. Photobiol.
C Photochem. Rev..

[43] Mao W. X., Lin X. J., Zhang W., Chi Z. X., Lyu R. W., Cao A. M., Wan L. J. (2016). Core-Shell
Structured TiO2@polydopamine
for Highly Active Visible-Light Photocatalysis. Chem. Commun..

[44] Guo Z., Wang G., Fu H., Wang P., Liao J., Wang A. (2020). Photocatalytic Degradation
of Methylene Blue by a Cocatalytic PDA/TiO2electrode
Produced by Photoelectric Polymerization. RSC
Adv..

[45] Zou Y., Chen X., Yang P., Liang G., Yang Y., Gu Z., Li Y. (2025). Regulating
the Absorption Spectrum of Polydopamine. Sci.
Adv..

[46] Kim Y., Coy E., Kim H., Mrówczyński R., Torruella P., Jeong D. W., Choi K. S., Jang J. H., Song M. Y., Jang D. J., Peiro F., Jurga S., Kim H. J. (2021). Efficient
Photocatalytic Production of Hydrogen by Exploiting the Polydopamine-Semiconductor
Interface. Appl. Catal. B Environ..

[47] Gautam S. K., Das A., Singh R. G., Kumar V. V. S., Singh F. (2016). Carrier Transport Mechanism
of Highly-Sensitive Niobium Doped Titanium Dioxide/p-Si Heterojunction
Photodiode under Illuminations by Solar Simulated Light. J. Appl. Phys..

[48] Dewasi A., Mitra A. (2018). UV–Visible Light
Detection with TiO2 Thin Film Deposited on
Chemically Textured p-Si Substrate. J. Mater.
Sci. Mater. Electron..

[49] Gengenbach T. R., Major G. H., Linford M. R., Easton C. D. (2021). Practical Guides
for X-Ray Photoelectron Spectroscopy (XPS): Interpreting the Carbon
1s Spectrum. J. Vac. Sci. Technol., A.

[50] Almeida L. C., Frade T., Correia R. D., Niu Y., Jin G., Correia J. P., Viana A. S. (2021). Electrosynthesis of Polydopamine-Ethanolamine
Films for the Development of Immunosensing Interfaces. Sci. Rep..

[51] Kubelka P., Munk F. (1931). A Contribution to the
Optics of Pigments. Z.
Technol. Phys..

[52] Makuła P., Pacia M., Macyk W. (2018). How To Correctly Determine the Band
Gap Energy of Modified Semiconductor Photocatalysts Based on UV-Vis
Spectra. Journal of Physical Chemistry Letters..

