# Supplementary material for: Heterojunction of Silicon Nanowires and TiO2 via Bioinspired Polymer for Efficient Photocatalytic Hydrogen Evolution
Source: ACS Appl Mater Interfaces. 2025 May 29;17(23):34504–12. doi: 10.1021/acsami.5c03529 (PMC12163933; doi:10.1021/acsami.5c03529)
Supplement: Supplementary file 1 [file am5c03529_si_001.pdf]

Supporting Information

Heterojunction of Silicon Nanowires and TiO_2 via Bioinspired Polymer for Efficient Photocatalytic Hydrogen Evolution

Jakub Szewczyk[1*], Stefanos Chaitoglou[2,3], Igor Iatsunskyi[1], Ghulam Farid[2,3], Mariusz Jancelewicz[1], Roger Amade-Rovira[2,3], Enric Bertran-Serra[2,3], Emerson Coy[1]

1 - NanoBioMedical Centre, Adam Mickiewicz University, Wszechnicy Piastowskiej 3, 61-614 Poznan, Poland

2 - Department of Applied Physics, University of Barcelona, C/Martí i Franquès, 1, 08028 Barcelona, Catalunya, Spain

3 - ENPHOCAMAT Group, Institute of Nanoscience and Nanotechnology (IN2UB), University of Barcelona, C/Martí i Franquès, 1, 08028 Barcelona, Catalunya, Spain

** Corresponding author: jakub.szewczyk@amu.edu.pl*

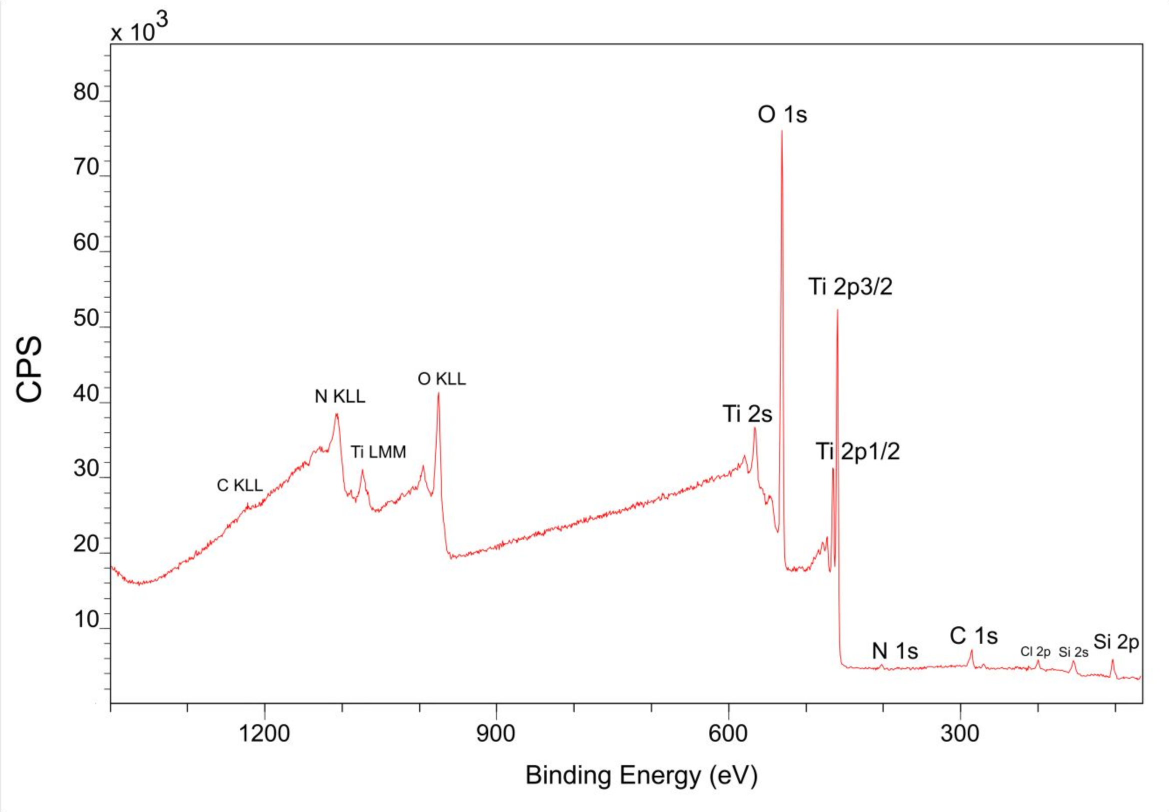

Figure S1. XPS full spectra of the SiNW/BAPDA/TiO_2 sample.

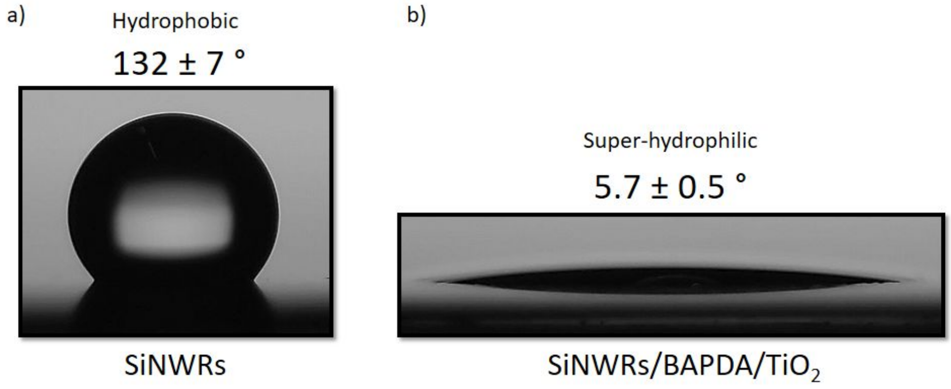

Figure S2. Water contact angle of the: a) hydrophobic surface of the SiNW, b) super-hydrophilic surface of the SiNW/BAPDA/TiO_2.

Table S1. EIS EEC fitting model and parameters

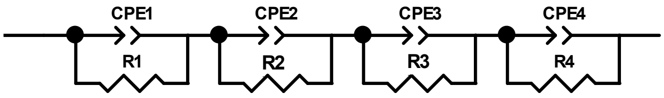

SiNW - dark<br>EEC fitting χ^2 = 0.0068	R1	89.6 ± 1.3 (Ω cm^{-2})	Q1	1.14 x 10^{-7} (F cm^{-2})
			n1	0.87 (-)
	R1	101 ± 7 (Ω cm^{-2})	Q2	2.03 x 10^{-4} (F cm^{-2})
			n2	0.71 (-)
	R3	90 ± 4 (Ω cm^{-2})	Q3	1.72 x 10^{-5} (F cm^{-2})
			n3	0.71 (-)
	R4	7000 ± 600 (Ω cm^{-2})	Q4	6.33 x 10^{-4} (F cm^{-2})
			n4	0.71 (-)

SiNW - light<br>EEC fitting χ^2 = 0.0053	R1	84 ± 1 (Ω cm^{-2})	Q1	1.14 x 10^{-7} (F cm^{-2})
			n1	0.88 (-)
	R1	84 ± 5 (Ω cm^{-2})	Q2	1.54 x 10^{-4} (F cm^{-2})
			n2	0.73 (-)
	R3	82 ± 3 (Ω cm^{-2})	Q3	1.67 x 10^{-5} (F cm^{-2})
			n3	0.71 (-)
	R4	9000 ± 700 (Ω cm^{-2})	Q4	5.13 x 10^{-4} (F cm^{-2})
			n4	0.70 (-)

SiNW/BAPDA/TiO_2 - dark<br>EEC fitting χ^2 = 0.0079	R1	48.9 ± 0.6 (Ω cm^{-2})	Q1	5.84 x 10^{-6} (F cm^{-2})
			n1	0.69 (-)
	R1	52 ± 2 (Ω cm^{-2})	Q2	3.72 x 10^{-4} (F cm^{-2})
			n2	0.70 (-)
	R3	250 ± 20 (Ω cm^{-2})	Q3	2.32 x 10^{-3} (F cm^{-2})
			n3	0.75 (-)
	R4	1800 ± 200 (Ω cm^{-2})	Q4	2.20 x 10^{-3} (F cm^{-2})
			n4	0.88 (-)

SiNW/BAPDA/TiO_2 - light<br>EEC fitting χ^2 = 0.0098	R1	46.5 ± 0.7 (Ω cm^{-2})	Q1	4.74 x 10^{-6} (F cm^{-2})
			n1	0.70 (-)
	R1	36 ± 2 (Ω cm^{-2})	Q2	3.26 x 10^{-4} (F cm^{-2})
			n2	0.69 (-)
	R3	33 ± 7 (Ω cm^{-2})	Q3	3.01 x 10^{-3} (F cm^{-2})
			n3	0.70 (-)
	R4	3900 ± 500 (Ω cm^{-2})	Q4	1.39 x 10^{-3} (F cm^{-2})
			n4	0.74 (-)

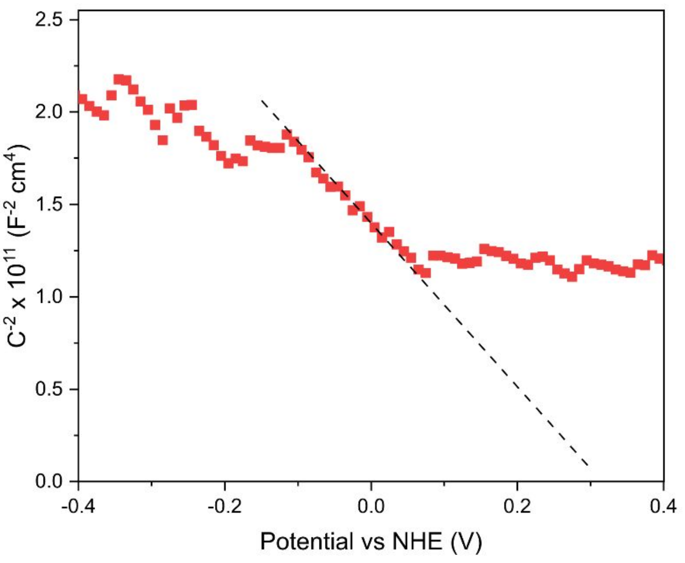

Figure S3. EIS Mott-Schottky analysis for the SiNW
